# Photonic Technology for In Vivo Monitoring of Hypoxia–Ischemia

**DOI:** 10.1002/advs.202204834

**Published:** 2022-11-15

**Authors:** Ion Olaetxea, Hector Lafuente, Eneko Lopez, Ander Izeta, Ibon Jaunarena, Andreas Seifert

**Affiliations:** ^1^ CIC nanoGUNE BRTA San Sebastián 20018 Spain; ^2^ Department of Communications Engineering University of the Basque Country Bilbao 48013 Spain; ^3^ Biodonostia Health Research Institute San Sebastián 20014 Spain; ^4^ Tecnun School of Engineering ‐ University of Navarra San Sebastián 20018 Spain; ^5^ IKERBASQUE Basque Foundation for Science Bilbao 48009 Spain

**Keywords:** hypoxia‐ischemia, machine learning, perinatal asphyxia, photonic monitoring, Raman spectroscopy

## Abstract

Surveillance of physiological parameters of newborns during delivery triggers medical decision‐making, can rescue life and health, and helps avoid unnecessary cesareans. Here, the development of a photonic technology for monitoring perinatal asphyxia is presented and validated in vivo in a preclinical stage. Contrary to state of the art, the technology provides continuous data in real‐time in a non‐invasive manner. Moreover, the technology does not rely on a single parameter as pH or lactate, instead monitors changes of the entirety of physiological parameters accessible by Raman spectroscopy. By a fiber‐coupled Raman probe that is in controlled contact with the skin of the subject, near‐infrared Raman spectra are measured and analyzed by machine learning algorithms to develop classification models. As a performance benchmarking, various hybrid and non‐hybrid classifiers are tested. In an asphyxia model in newborn pigs, more than 1000 Raman spectra are acquired at three different clinical phases—basal condition, hypoxia–ischemia, and post‐hypoxia–ischemia stage. In this preclinical proof‐of‐concept study, figures of merit reach 90% levels for classifying the clinical phases and demonstrate the power of the technology as an innovative medical tool for diagnosing a perinatal adverse outcome.

## Graphical abstract

Given the shortcomings of state‐of‐the‐art perinatal monitoring, the combination of Raman spectroscopy with machine learning is evaluated in vivo as an innovative diagnostic tool. The great performance achieved for identification of hypoxic–ischemic events confirms this technology as a credible alternative for supporting medical decision‐making by non‐invasive, continuous, and real‐time monitoring of perinatal asphyxia.

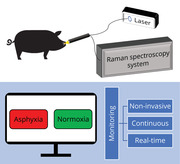

## 1 Introduction

Worldwide, the number of newborn deaths counts for 2.5 million, of which 24% are caused by intrapartum complications such as perinatal asphyxia, one of the most common medical disorders in high‐risk births.^[^ ^1^ ^]^ Characterized by insufficient oxygen supply to fetal tissue, a prolonged episode of perinatal asphyxia can produce neurological damage known as hypoxic–ischemic encephalopathy.^[^ ^2^, ^3^ ^]^ Fetal monitoring plays a major role in the diagnosis of perinatal asphyxia, and consequent neonatal adverse outcomes. However, performance of current standard methods, such as cardiotocography (CTG) or fetal scalp blood sampling (FSBS) have been questioned in obstetric care.

CTG, which registers the fetal heart rate and uterine contractions, has been identified as one of the multiple factors responsible for the increase of cesarean sections and instrumental deliveries.^[^ ^4^, ^5^ ^]^ Apart from outcomes of CTG monitoring, rising voluntary cesareans constitute a major issue that strongly depends on country and culture. The average rate of cesareans worldwide is ≈22%, which is far above the recommended value of 15% by the World Health Organization (WHO).^[^ ^6^ ^]^ Moreover, the WHO states that caesarean section rates >10% are not associated with reductions in maternal and newborn mortality rates.^[^ ^7^ ^]^

FSBS is a complementary technique to detect perinatal asphyxia and reduce the number of unnecessary cesareans. By a small invasive incision, blood is extracted from the scalp and generally analyzed by blood gas analysis. The clinical diagnosis is performed according to the pH or, alternatively, the concentration of lactate.^[^ ^8^, ^9^ ^]^ The method, which operates in an invasive, discontinuous and intermittent fashion, does not provide a real picture of the clinical status of the fetus, leading to a high misclassification rate.^[^ ^9^, ^10^ ^]^

Spectroscopic techniques are constantly gaining ground in medical diagnostics due to their great capabilities to provide valuable information regarding the interaction of light with matter at molecular level.^[^ ^11^, ^12^ ^]^ Near‐infrared spectroscopy or microwave sensors have been considered as credible attractive alternatives for non‐invasive monitoring of pH and lactate.^[^ ^13^, ^14^ ^]^ However, apart from not showing enough predictive accuracy or reliability for clinical use, the authors have previously demonstrated that a diagnosis based on a systemic physiological picture, represented by the entirety of biochemical parameters, yields a substantial improvement in the predictive performance over current diagnosis based on pH or lactate.^[^ ^15^ ^]^

In this study, a new disruptive and powerful diagnostic tool for monitoring perinatal asphyxia is presented, employing Raman spectroscopy in combination with machine learning. Such a combination has become of general interest in medical research as it has successfully been applied in different fields like food control,^[^ ^16^ ^]^ tumor margin identification,^[^ ^17^ ^]^ analysis of viruses and bacteria,^[^ ^18^, ^19^ ^]^ or sensing of body fluids.^[^ ^20^ ^]^ The technology represents a photonic non‐invasive approach that works in continuous mode, delivering real‐time diagnostics. We tested and validated our method in vivo in an animal model with newborn piglets. The method is not limited to examine a single parameter as pH or lactate, but rather considers the entirety of physiological parameters that are accessible by vibrational spectroscopy, and hence, delivers a systemic picture measured at molecular level. Identification of physiological anomalies related to perinatal asphyxia will support immediate medical decision‐making in an innovative way.

## 2 Results

**Figure 1:**
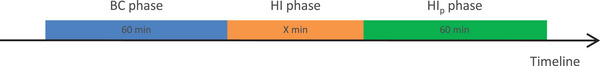
Experimental timeline divided in three phases: basal condition phase BC, 60 min before the damage phase; hypoxic–ischemic damage phase HI, variable time until one or more of the established thresholds are reached; post‐hypoxia–ischemia phase HI_p_, 60 min after the damage phase.

In total, three phases were established throughout the experiment, as shown in **Figure** **1**. While basal condition (BC) and post‐hypoxia–ischemia (HI_p_) phases were defined to last 60 min, the duration of the hypoxia–ischemia (HI) phase was variable as the time of reaching the end‐point criterion was different in each experiment. The mean duration of the HI damage was 49 ± 15 min.

### 2.1 pH and Lactate as Gold Standard Parameters

By inducing an HI event, all measured physiological parameters change their value, more or less pronounced, compared to basal condition, which is reflected in Figure S1 (Supporting Information). These findings support the conclusions drawn by Lafuente et al.^[^ ^15^ ^]^ and confirm our initial hypothesis that considering systemic physiological variations will provide a much better predictive model. The Mann–Whitney *U* test unveils statistical significance for all parameters, including O_2_ and pO_2_, both for HI (Table S1, Supporting Information) and HI_p_ (Table S2, Supporting Information) conditions when compared to BC. However, as the sample size is relatively large, the p‐value might not be appropriate to assume significant differences of the observables.^[^ ^21^ ^]^ For large datasets, the p‐value of a statistical test is very likely to be significant even for minor variations. In such cases the effect size represents a more appropriate quantity that describes the real magnitude of the differences and is reported by the rank‐biserial correlation. The simplified coefficient proposed by Wendt et al.^[^ ^22^ ^]^ and defined as

(1) $$\mathrm{Effect}\;\mathrm{size}=1-\frac{2U}{n_{1}n_{2}}$$

takes values between 0, for no correlation, and 1, for maximum correlation, where *U* is the test statistics, and *n*_1_ and *n*_2_ are the group sizes. Even though the effect size has considerable magnitudes for all parameters during HI (Table S1, Supporting Information), interestingly it is negligible in the HI_p_ period for the oxygen‐related parameters SO_2_ and pO_2_ (Table S2, Supporting Information).

Parameters such as lactate, pH, base excess, TCO_2_ or HCO${}_{3}^{-}$ show a much slower recovery to initial values, when ventilation is restored after a severe HI event. Accordingly, these parameters show strong correlation with the clinical state (BC, HI, and HI_p_), resulting in an absolute Spearman's rank correlation coefficient close to 0.8. While lactate exhibits a positive correlation with the occurrence of an HI event, the rest of relevant parameters shows a negative correlation (**Table** **1**).

**Table 1:** Spearman's rank correlation coefficient ρ between physiological parameters and the clinical state associated with the established phases BC, HI, HI_p,_ and corresponding statistical significance given by the p‐value

Parameter	ρ	p‐value
Lactate	0.81	<0.001*
pCO_2_	0.33	<0.001*
SO_2_	0.01	0.84
pO_2_	−0.10	<0.001*
pH	−0.76	<0.001*
TCO_2_	−0.81	<0.001*
Base excess	−0.81	<0.001*
HCO${}_{3}^{-}$	−0.81	<0.001*

Binary classification of BC and HI conditions for clinical pH and lactate cut‐off limits is displayed in Figure S2 (Supporting Information). The figures of merit in **Table** **2** summarize their classification performance. Lactate demonstrates to be a reasonably better indicator of an HI event than pH. Likewise, the little capacity of current standards to detect positive cases provides a poor sensitivity of 55.7% for pH and 72.1% for lactate. The reason for this poor outcome is the time lag between the beginning of the HI event and the moment when clinical standard cut‐off limits are exceeded.

**Table 2:** Classification performance of pH and lactate standard cut‐off limits for differentiation of BC and HI conditions; all figures of merit are in percentage (%)

	pH < 7.2	[Lactate] > 4.8 mM
Sensitivity	55.7	72.1
Specificity	100	100
Accuracy	78.6	86.5
AUROC	77.9	86.1

AUROC: Area under the receiver operating characteristics.

Performance of pH and lactate cut‐off limits improves for binary classification between BC and HI_p_ conditions due to a considerable increase of their sensitivity, as shown in **Table** **3**. As the HI phase is not considered, the delay between the beginning of the HI phase and the moment when cut‐off limits are exceeded is not reflected in the biochemical data from blood gas analysis. Instead, biochemical values corresponding to HI_p_ condition already start far beyond established pH and lactate thresholds. Moreover, inducing severe HI results in a slow recovery to basal condition, which contributes to a greater class separability in terms of AUROC (area under the receiver operating characteristics) and, consequently, a better classification performance (Figure S3, Supporting Information).

**Table 3:** Classification performance of pH and lactate standard cut‐off limits for differentiation of BC and HI_p_ conditions; all figures of merit are in percentage (%)

	pH < 7.2	[Lactate] > 4.8 mM
Sensitivity	79.7	95.9
Specificity	100	100
Accuracy	89.6	97.9
AUROC	89.8	97.9

When all three phases are considered, prediction based on univariate analysis of a single parameter, such as pH or lactate, is not feasible since additional information or definitions for differentiating these three classes do not exist. Apart from established cut‐off limits addressing HI, no further distinction is done among the groups. As a result, an overlap between HI and HI_p_ is observed in Figure S4 (Supporting Information).

### 2.2 Predictive Models Based on Raman Spectra

The combination of Raman spectroscopy with machine learning algorithms is tested as an alternative to current gold standard method. The performance of different predictive models is evaluated for the classification problems stated above. Typical preprocessed Raman spectra of all three clinical states are shown in Figure S5 (Supporting Information).

#### 2.2.1 Basal Condition versus HI

For the different classifiers used for differentiation of BC and HI phases, **Table** **4** displays the corresponding figures of merit as above in the case of univariate analysis. In this kind of benchmarking, the following classifiers have been employed: partial least squares – discriminant analysis (PLS–DA), random forest (RF), support vector machine (SVM), adaptive boosting (AB), gradient boosting (GB), and extreme gradient boosting (XGB). Apart from SVM, which exhibits an accuracy of 85.4%, all predictive models present an accuracy > 87.4%, which is slightly superior to the performance achieved by the cut‐off limit of lactate; regarding pH, the difference in performance is even larger. The substantial improvement of the sensitivity ( > 88%) achieved by the predictive models constitutes the major variation in the classification of BC and HI phases.

**Table 4:** Classification performance of different machine learning algorithms for differentiation of (BC) and (HI) phases; all figures of merit are in percentage (%)

	PLS–DA	RF	SVM	AB	GB	XGB
Sensitivity	89.5	88.9	88.5	88.2	89.5	90.4
Specificity	86.4	86.1	82.4	87.6	89.0	89.0
Accuracy	87.9	87.4	85.4	87.9	89.2	89.7
AUROC	94.7	93.7	93.4	92.5	94.9	95.4

To understand how different algorithms differentiate BC and HI phases, the accuracy decrease, obtained by feature permutation of PLS–DA and XGB predictive models, is displayed in Figure S6a,b (Supporting Information). Raman features are significant when the prediction error increases subject to shuffling these features. While PLS–DA, which maximizes the covariance between the matrix of observation and the matrix of outcomes, gets more or less affected by every feature permutation, XGB algorithm demonstrates to be much more robust to variations.

According to Figure S6a (Supporting Information), the PLS–DA predictive model identifies a number of relevant Raman features in the region from 1100 to 1650 cm^−1^ that arise from Raman‐active blood components, such as proteins or heme groups.^[^ ^23^ ^]^ Prominent peaks at 1222 and 1547 cm^−1^ have already been assigned to oxygenated and deoxygenated red blood cells, respectively.^[^ ^23^, ^24^, ^25^ ^]^ Interestingly, bands associated to lactate at 853 cm^−1^ from a C–C aliphatic stretching, 1053 cm^−1^ from C–CH_3_ vibration, or 1420 cm^−1^ from COO^−^ stretching are also observed. The Raman band ≈ 543 cm^−1^ describes probably the overlap of bands associated to the CO${}_{2}^{-}$ wagging of lactate and Fe–O_2_ stretching of hemoglobin. The assignment of prominent bands is summarized in **Table** **5**.

**Table 5:** Raman bands and their corresponding assignment to vibrational modes and molecules

Raman shift [cm^−1^]	Vibrational mode	Molecule
543	CO2${}_{2}^{-}$ wagging	Lactate
571	Fe‐O_2_ stretching	Hemoglobin
620	C‐C twisting	Protein denaturation
853	C‐C aliphatic stretching	Lactate
1005	Phenylalanine breathing	Protein
1053	C‐CH_3_ stretching	Lactate
1124	CH_3_ rocking & C‐O vibration	Lactate
1127	C_β_‐methyl vibration	Hemoglobin
1222	CH bending	Hemoglobin
1420	COO^−^ stretching	Lactate
1459	CH_3_ deformation	Lactate
1547	C_β_C_β_ stretching	Hemoglobin
1639	C_α_C_m_ asymmetric stretching	Hemoglobin

**Figure 2:**
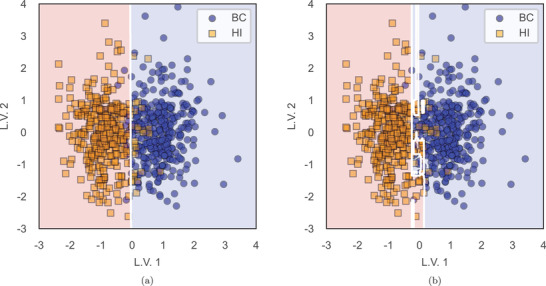
Example of 2D visual class separability between BC and HI phases by hybrid classification models optimized by a leave‐one‐subject‐out cross‐validation. a) Binary classification of BC versus HI based on the decision region defined by an SVM classifier in a PLS 2‐component dataset. b) Binary classification of BC versus HI based on the decision region defined by an RF classifier in a PLS 2‐component dataset.

In the case of PLS‐based hybrid classifiers, similar predictive power is achieved. The accuracy of non‐hybrid classifiers reaches values of 85.4 – 89.7%, while hybrid classifiers lie within the range of 85.2 87.7%, see **Table** **6**. Interestingly, figures of merit of PLS–SVM even improve compared to its usage alone. As previously mentioned, PLS is applied twice such that the information from relevant features is represented by only two PLS latent vectors. Applying PLS a second time should not affect the data and exclude relevant information. Consequently, the performance has been compared to single use of PLS first, where the classifier is built in a latent space formed by the optimum number of latent vectors. It has been observed that applying PLS twice does not result in any significant variation in the performance. **Figure** **2**a,b illustrates the decision region of SVM and RF classifiers applied in one step considering the LOSOCV (leave‐one‐subject‐out cross‐validation) method for training, which is explained in the Experimental Section below.

**Table 6:** Classification performance of different hybrid machine learning algorithms for differentiation of BC and HI phases; all figures of merit are in percentage (%)

	PLS‐RF	PLS–SVM	PLS–AB	PLS–GB	PLS–XGB
Sensitivity	88.9	89.5	87.0	87.3	88.2
Specificity	83.5	86.1	85.8	83.2	83.2
Accuracy	86.1	87.7	86.4	85.2	85.7
AUROC	92.5	94.7	93.9	92.6	93.6

#### 2.2.2 Basal Condition versus HI_p_

After restoring ventilation, blood oxygenation recovers quickly to basal level, and it becomes challenging to identify the HI event, which is reflected in the figures of merit obtained by different classifiers for differentiation of BC and HI_p_ phases, as listed in **Table** **7**. The predictive power to detect physiological changes caused by HI events decreases as time passes. In our experiments, the accuracy for HI_p_ lies within the range of 68.9 – 74.4%.

**Table 7:** Classification performance of different machine learning algorithms for differentiation of BC and HI_p_ phases; all figures of merit are in percentage (%)

	PLS–DA	RF	SVM	AB	GB	XGB
Sensitivity	80.8	70.1	77.5	76.6	76.9	78.6
Specificity	67.6	67.6	68.8	66.2	67.9	67.6
Accuracy	74.4	68.9	73.2	71.5	72.5	73.2
AUROC	82.5	75.3	81.6	77.5	78.8	79.6

It turns out that PLS–DA and XGB have the best performance among the classifiers (Table 7); corresponding Raman feature importance is calculated by the permutation importance technique and displayed in Figure S7a,b (Supporting Information). In the PLS–DA model, the contribution of the region from 1100 to 1650 cm^−1^ to identify the HI_p_ phase is reduced. Contributions from 1222 and 1547 cm^−1^ associated with blood oxygenation are still present. A new band ≈ 620 cm^−1^ is assigned to protein denaturation caused by the HI event, while the contribution at 1125 cm^−1^ might be concurrently provoked by lactate and hemoglobin. The peak at 1639 cm^−1^ is a marker for hemoglobin oxygenation and the prominent contribution of phenylalanine band from proteins ≈1005 cm^−1^ is also observed.^[^ ^23^, ^24^, ^25^ ^]^ Moreover, contribution of peaks associated to lactate at 543 cm^−1^, but mainly at 853 cm^−1^, is evident. In the case of XGB model, the importance of lactate is even more pronounced, and only permutations at 853 and 1459 cm^−1^ produce a decrease in the prediction accuracy.

**Figure 3:**
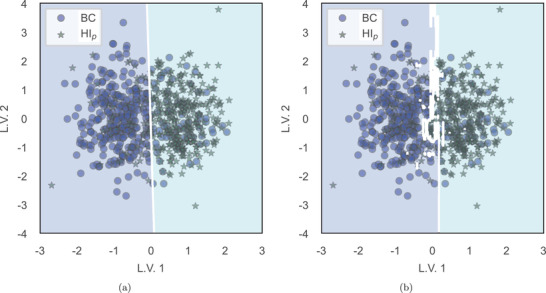
Example of 2D visual class separability between BC and HI_p_ phases by hybrid classification models optimized by a leave‐one‐subject‐out cross‐validation. a) Binary classification of BC versus HI_p_ based on the decision region defined by an SVM classifier in a PLS 2‐component dataset. b) Binary classification of BC versus HI_p_ based on the decision region defined by an RF classifier in a PLS 2‐component dataset.

In contrast to the HI phase, all hybrid predictive models show improved predictive power compared to non‐hybrid models in case of the differentiation of HI_p_. After restoring ventilation and partial recovery of physiological parameters to basal levels, the similarity of BC and HI_p_ Raman curves represents a challenge in the identification of HI events and the development of corresponding classification models. Dimensionality reduction serves here as useful data preprocessing step for complex data. **Figure** **3**a,b give examples of the decision region of SVM and RF classifiers for differentiating BC and HI_p_ states (**Table** **8**).

**Table 8:** Classification performance of different hybrid machine learning algorithms for differentiating BC and HI_p_ phases; all figures of merit are in percentage (%)

	PLS‐RF	PLS–SVM	PLS–AB	PLS–GB	PLS–XGB
Sensitivity	80.2	81.3	81.9	81.9	81.3
Specificity	69.9	67.9	68.8	69.7	69.1
Accuracy	75.2	74.8	75.5	75.9	75.4
AUROC	82.1	82.4	79.8	80.1	80.0

#### 2.2.3 Basal Condition versus HI versus HI_p_

Predictive models based on multiparametric data such as Raman spectra lead to a better representation of the clinical picture. Raman bands provide additional information associated to systemic physiological variations, which allows us to classify all different phases regarding an HI event. Figures of merit of different classifiers for differentiation of BC, HI, and HI_p_ phases are displayed in **Table** **9**. All of them present a total accuracy ≈ 80%, which is similar to that obtained for differentiation of BC and HI_p_ only and represents a remarkable outcome considering the similarities between groups.

**Table 9:** Classification performance of different machine learning algorithms for multiclass differentiation of BC, HI, and HI_p_ phases; all figures of merit are in percentage (%)

	PLS–DA	RF	SVM	AB	GB	XGB
Sensitivity	73.0	68.6	72.3	68.1	71.6	71.8
Specificity	86.3	84.0	85.9	83.7	85.6	85.6
Accuracy	81.9	78.8	81.3	78.5	80.9	81.0

Consequently, the permutation feature importance of PLS–DA in Figure S8a (Supporting Information) is a combination of those obtained for differentiating HI (Figure S6a, Supporting Information) and HI_p_ (Figure S7a, Supporting Information) independently. Thus, Raman bands at 543, 571, 620, 853, 1005, 1053, 1125, 1222, 1420, 1459, 1547, and 1639 cm^−1^ are identified as relevant for detecting HI events and classification of BC, HI, and HI_p_ phases. In contrast, XGB is only affected by permutations at 1547 cm^−1^.

**Figure 4:**
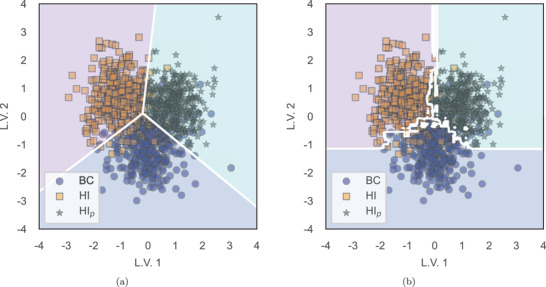
Example of 2D visual multiclass separability between BC, HI, and HI_p_ phases by hybrid classification models optimized by a leave‐one‐subject‐out cross‐validation. a) Multiclass classification of BC versus HI versus HI_p_ based on the decision region defined by an SVM classifier in a PLS 2‐component dataset. b) Multiclass classification of BC versus HI versus HI_p_ based on the decision region defined by an RF classifier in a PLS 2‐component dataset.

Hybrid classifiers have demonstrated to yield predictive power similar, and even slightly better in some cases, to non‐hybrid ones; corresponding figures of merit are depicted in **Table** **10**. As stated before, a big advantage of hybrid predictive models is the option to graphically illustrate the decision region defined by different classifiers. **Figure** **4**a,b are explicitly meaningful representations of a multiclass classification, where BC, HI, and HI_p_ phases are differentiated based on their Raman spectra by PLS–SVM and PLS‐RF classifiers, respectively.

**Table 10:** Classification performance of different hybrid machine learning algorithms for multiclass differentiation of BC, HI, and HI_p_ phases; all figures of merit are in percentage (%)

	PLS‐RF	PLS–SVM	PLS–AB	PLS–GB	PLS–XGB
Sensitivity	72.2	73.2	72.8	72.2	71.7
Specificity	85.9	86.4	86.2	85.9	85.6
Accuracy	81.3	82.0	81.7	81.3	81.0

## 3 Discussion

Apart from operating in an invasive manner, fetal scalp blood sampling (FSBS) has proven limited evidence for accurate identification of perinatal asphyxia and produces considerable false positive and false negative rates. The shortcomings of state of the art call for new diagnostic tools that overcome the limitations. Our technology, which combines Raman spectroscopy with machine learning, exhibits great potential for more accurate and robust monitoring of perinatal asphyxia.

Statistical tests, such as Mann–Whitney *U* test or Spearman's correlation, have unveiled significant changes of a number of physiological parameters upon HI. Following the gold standard technique, pH and lactate have been used as decision‐making parameter, but both of them present difficulties in detecting HI events shortly after its occurrence due to the delay that exists from the insult until exceeding cut‐off limits. Prolonged HI results in extreme pH and lactate values and consequently, in a slow recovery to basal state. Therefore, the predictive power considerably increases for differentiating HI_p_ phase, particularly with lactate as indicator, which generally proves to be the better indicator than pH. It is important to point out that this outcome is obtained as a result of a severe HI event extended over almost 50 min. Another big issue in the diagnosis based on a single parameter is the lack of valuable information to differentiate HI and HI_p_ phases. Distinction between these two stages will be a great asset in medical assessment.

As an alternative, we applied Raman spectroscopy extracorporeally to living animals, and we consider the entirety of physiological parameters that are accessible by vibrational spectroscopy; the Raman spectra are then analyzed by several machine learning algorithms. Predictive models have demonstrated great performance for the identification of HI events. Even though their predictive capacity is reduced at HI_p_, all models achieve excellent discriminating power during the HI phase, even better to that obtained by lactate or pH from blood gas analysis. No significant differences have been found between different algorithms, but overall, PLS–DA and XGB have shown slightly better performance. Efficiency of PLS has widely been confirmed for functional data, i.e., curve or spectrum type problems, where the dimension of the observation space—broad spectral range—includes multicollinearity of features. The potential of XGB for classification problems is beyond doubt, since XGB is regularly winning special classification competitions. Nonetheless, each model has its strength and makes its own interpretation of data. The permutation feature importance technique allows us to understand how the model is constructed and, consequently, guarantees a meaningful classification. Raman bands associated to red blood cell oxygenation—such as heme groups—or protein denaturation, have been identified as main contributors for classification of HI. In line with Lafuente et al.,^[^ ^15^ ^]^ lactate is also recognized as a suitable biomarker, especially at HI_p_, when ventilation and oxygenation is already restored.

As stated above, PLS‐based hybrid classifiers allow for graphical representation, as Raman data is projected to a new space of latent vectors. As a result, decision regions defined by different classifiers can be easily compared. Moreover, hybrid classifiers have demonstrated to achieve similar, or in some cases even better, performance than non‐hybrid ones. Given the ability of PLS to deal with multicollinearity, applying classifiers after extraction of relevant features strongly supports successful classification.

**Figure 5:**
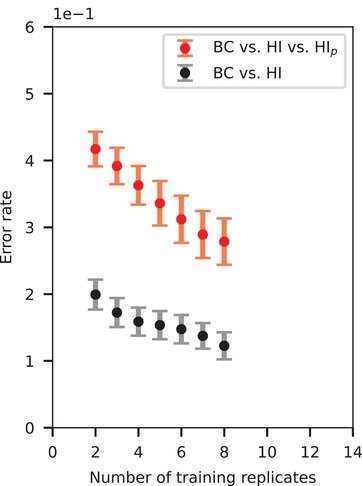
Progression of the error rate as a function of the training sample size. Averaged learning curve and its standard error for BC versus HI classification and BC versus HI versus HI_p_ multiclass classification.

So far, the number of subjects in this study is not very high, however, the number of reliable Raman spectra from all three clinical stages—basal condition, HI, HI_p_—represents a reasonable statistics. Achieved results exhibit great potential of our technology for HI monitoring and is supported by a decreasing trend of the learning curve of the PLS–DA model for classification problems (**Figure** **5**). This learning curve is calculated as the average of individual learning curves belonging to one subject, and is represented by the classification error rate as a function of the number of training replicates.^[^ ^26^ ^]^ In other words, for a given number of training replicates, a piglet is selected for validation, while the rest is used to form all possible permutations. Classifiers that have been constructed from each of those training sets are then tested in the validation piglet and the error rate is averaged. This procedure is repeated for each piglet replicate, and the mean together with the standard error of individual learning curves are depicted in Figure 5. It is important to emphasize that this study has been carried out with a commercial Raman probe (thRamanProbe^TM^, InPhotonics)^[^ ^27^ ^]^ that despite of an application‐specific modification has not been designed for this specific task. So far, applicability of the technology has been proven in a well‐controlled environment. The implementation of the technology to real clinical settings has to consider unexpected interferences and uncertainties directly related to childbirth. The development of an optomechanically optimized probe that faces real‐life situations is realistically feasible and will result in considerable improvement of the performance.

## 4 Conclusion

For many years, obstetric care is relying on pH from fetal scalp blood sampling as a standard biomarker for fetal surveillance. The limited capacity to avoid adverse neonatal outcomes and cesarean deliveries gave lactate, in vain, the opportunity to be considered as a more suitable alternative. It has been demonstrated that HI is responsible for an overall impact in the metabolism, which is reflected in systemic variations of different physiological parameters. The ability to non‐invasively provide high molecular selectivity makes Raman spectroscopy an ideal method for clinical assessment. This study presents the combination of Raman spectroscopy with machine learning as a potential diagnostic tool for monitoring perinatal asphyxia. Apart from exhibiting great performance for identifying a HI event, predictive models based on Raman spectra perform relevant distinction between damage (HI) and recovery (HI_p_) phases. In conclusion, the technology represents an innovative approach that will support immediate medical decision‐making by non‐invasive, continuous and real‐time monitoring of perinatal asphyxia.

## 5 Experimental Section

### Animal Preparation

Nine piglets, 1–3 days old, were anesthetized with sevoflurane (inducing 5% and maintenance 2.5%) and fentanyl (0.0002 – 0.0005 mg kg^−1^ h^−1^), and then paralyzed by a perfusion of vecuronium (1.8 mg kg^−1^ h^−1^) administered through the ear vein. Animals were then intubated and ventilated with positive pressure. The femoral artery was cannulated to monitor blood pressure and to obtain blood samples. Blood oxygen saturation was monitored by transcutaneous pulse oximetry.

After stabilization, the animals were kept under normoxic conditions for 1 h (BC phase). Inducing HI damage was carried out by reducing the inspiratory oxygen fraction between 8 – 10% until reaching a base excess (BE) ⩽ −15 mEq L^−1^ and/or a pH ⩽ 7 and/or a lactate concentration ⩾ 12 mM (moderate‐severe HI damage). In addition, a mean arterial pressure < 20 mmHg was established as an end‐point criterion for hypoxia, since in these cases, the life of the animal is seriously compromised. To reduce the inspiratory fraction of oxygen below 21%, the ventilation system was modified, replacing the medical air supply with nitrogen and readjusting oxygen concentrations with an oximeter. In addition, CO_2_ was administered in the inspiratory fraction (PaCO_2_ 8.0 – 9.5 kPa), with the aim to mimic the perinatal asphyxia (HI) phase. After hypoxic damage, the inspired oxygen fraction was restored to baseline levels and kept for 1 h (HI_p_ phase).

For seven of the piglets, the experimental procedure was carried out in accordance with the established protocol and no difficulties were reported. The piglet with replicate ID n° 6 suffered a cardiac arrest during the HI phase after 19 min of damage and fulfillment of end‐point criterion. Despite this unexpected setback, the experiment could be completed after resuscitation. Similarly, the piglet with replicate ID n° 9 suffered a cardiac arrest. In that case, the heart failure happened when normal oxygenation was restored, and the experiment could not be completed since we did not succeed in resuscitation. Therefore, there is no available data from HI_p_ phase for the piglet with replicate ID n° 9.

At the end of the experiment, the piglets were euthanized with an intravenous injection of potassium chloride. All experimental procedures and euthanasia of the animals were conducted in strict compliance with European and Spanish regulations on the protection of animals used for scientific purposes (European Directive 2010/63/EU and Spanish Royal Legislative Decree 53/2013). The protocols were approved by the Committees on the Ethics of Laboratory Animal Welfare of Biodonostia Health Research Institute (Permit Numbers: OH 18_22 and OH 20_36) and performed in its experimental surgical theater.

### Blood Gas Analysis

Throughout the experiment, blood samples were extracted from femoral artery for gas analysis with the i‐STAT 1 handheld blood analyzer (Abbott Laboratories). **Table** **11** shows the eight physiological parameters that were provided by the gas analayzer. Due to the amount of time required for the whole extraction and analysis procedure, blood samples were extracted every 10 Raman measurements. Nonetheless, in order to match each Raman measurement with a blood gas analysis, a linear equation was fitted in every two points for each physiological parameter, and values in between assigned to their corresponding spectrum. Thus, a parallel database with 1033 blood gas analyses was additionally generated. This second database was divided in three subsets, normoxia or basal condition phase (BC, *n* = 346), HI phase *(n* = 323) and HI_p_ phase *(n* = 364). The numbers are the numbers of corresponding Raman spectra (see next subsection).

**Table 11:** Physiological parameters measured by blood gas analysis

pH	pCO_2_ [mmHg]	pO_2_ [mmHg]	Base excess [mEq/L]
[6.88 – 7.55]	(30.3 – 85.0)	(14 – 608)	(−20 – 13)
HCO${}_{3}^{-}$ [mmHg]	TCO_2_ [mmHg]	SO_2_ [mmHg]	Lactate [mM]
(11.9 – 37.2)	(13 – 39)	(13 – 100)	(0.41 – 14.2)

### Raman Data Acquisition

**Figure 6:**
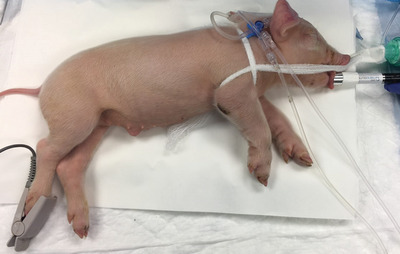
Experimental setup for in vivo non‐invasive monitoring of hypoxia–ischemia (HI) by Raman spectroscopy in piglets. HI damage is induced by reducing the inspired oxygen fraction. The RamanProbe^TM^ is placed at the tongue and blood samples are extracted from femoral artery for further blood gas analysis.

In the current study, both Raman spectra and physiological parameters were simultaneously acquired. Raman measurements were performed with the RamanProbe^TM^ (RPS785, InPhotonics), which had been adapted to be used in contact with the skin and optimally placed laser focus. The RamanProbe^TM^ with an excitation fiber of 105 µm core and a collection fiber of 200 µm is connected to a continuous wave diode laser, emitting at 785 nm, and to the EAGLE Raman‐S grating spectrometer with Andor iVac 316 detector (Ibsen Photonics) with a spectral resolution of 4 cm^−1^. Due to its suitable Raman signal and high level of irrigation, the tongue was used as excitation tissue for identification of HI events (**Figure** **6**). Even though Raman spectra were continuously collected, for each measurement the probe was replaced to a different position to avoid any possible bias or sample damage caused by long and permanent exposures. Integration time was 60 s at a power of 60 mW. In total, 1033 Raman spectra were measured and analyzed. The database consists of three subsets, normoxia or BC phase (*n* = 346), HI phase (*n* = 323) and HI_p_ phase ( = 364).

### Univariate Analysis

Physiological parameters depicted in Table 11 have been assessed individually. Box‐and‐whisker plots were used to depict data distribution. Non‐parametric Mann–Whitney *U* test was applied to determine statistical significance among groups. Used as an alternative to the independent sample t‐test, Mann–Whitney *U* test does not assume normal distribution of the dependent variable and the test statistic is calculated as a function of the rank difference rather than mean difference. Spearman's rank correlation is used to measure the monotonic relationship between physiological parameters and the clinical state. Finally, predictive power of lactate and pH for classification of HI was examined by means of statistical metrics derived from a confusion matrix, including sensitivity, specificity, accuracy, and the area under the receiver operating characteristic curve (AUROC). Again, definition of perinatal asphyxia was based on clinical cut‐off limits of pH < 7.20 and [lactate] > 4.8 mM as proposed by Saling and Kruger et al.,^[^ ^28^, ^29^ ^]^ respectively.

### Preprocessing of Raman Spectra

Analysis of Raman data from biological samples is extremely challenging due to their heterogeneous nature. Moreover, it is even more complex and difficult to handle in vivo data. Therefore, prior to perform any statistical analysis or develop any predictive model, preprocessing of raw Raman data is essential for the correct interpretation of the Raman spectra and for a reliable classification of the samples.

Accordingly, all Raman spectra were first trimmed to the spectral range of 535 to 1717 cm^−1^, the so‐called fingerprint region. Then, a 6th order Extended Multiplicative Signal Correction (EMSC) was applied to the raw spectra to remove interfering additive and multiplicative artifacts by scaling all the Raman spectra to the mean spectrum.^[^ ^30^ ^]^ An asymmetric least squares (ALS) method, based on Whittaker filter, was used to subtract a smoothed background produced by the intrinsic fluorescence of the molecules.^[^ ^31^ ^]^ The integrity of the Raman bands is retained by giving higher relevance to positive residuals.^[^ ^32^ ^]^ All Raman spectra were smoothed using a Savitzky–Golay filter with a window of 15 points and 3rd order polynomial at a sampling of 4 cm^−1^. Finally, to avoid uncertainties of the variance of the mean, the origin of the coordinate system is translated to the center of gravity of the dataset. Some machine learning algorithms are built upon the variance of specific features of data, and then it is important to center the data relative to a reference point, which in this case is the mean. Combination of all data preprocessing has been optimized based on maximization of Raman features and performance of the predictive models.

Even though the experiments were carried out in a well‐controlled experimental context, in vivo measurements imply a considerable degree of uncertainty. In order to avoid the presence of outliers in the database, a principal component analysis (PCA) is individually applied to the Raman data of each animal. Outliers are identified based on a Q residuals vs. Hotteling's *T* ^2^ plot. Such a plot represents the lack‐of‐fit and the captured variation of each sample within the model. Samples out of the confidence limit of 95% were not considered and were discarded from proceeding data analysis. All data preprocessing was carried out with the PLS_Toolbox (Eigenvector) in MATLAB environment (MathWorks).

### Multivariate Analysis

It is well‐known that a hypoxic‐ischemic event induces a physiological response, which has been demonstrated to cause a generalized systemic reaction rather than the variation of a single biomarker.^[^ ^15^ ^]^ Such a physiological response is reflected in distinct spectral features of Raman bands. However, identification and assignment of peaks, and consequently, differentiation among groups constitutes a serious challenge, which becomes even more complex with the classification of in vivo data. A number of different classification algorithms—as single classifier, ensemble learning and hybrid classifiers—were used for the detection of HI. Specifically, i) partial least squares – discriminant analysis (PLS–DA),^[^ ^33^ ^]^ ii) random forest (RF),^[^ ^34^ ^]^ iii) support vector machine (SVM),^[^ ^35^ ^]^ iv) adaptive boosting (AB),^[^ ^36^ ^]^ v) gradient boosting (GB),^[^ ^36^, ^37^ ^]^ and vi) extreme gradient boosting (XGB)^[^ ^38^ ^]^ were tested. Fundamental concepts of applied algorithms can be found in more detail in the Supporting Information.

Relevant spectral bands for identification and classification of the clinical state, associated to BC, HI, and HI_p_, were determined. Their contribution to different predictive models was calculated by the permutation importance technique. The method calculates the decrease of a statistical metrics of a fitted model when a single feature is randomly shuffled. In other words, the accuracy decrease of each optimized predictive model was calculated when a single wavenumber was randomly shuffled. The process was repeated for every wavenumber. The magnitude of the decrease reveals how decisive that spectral feature is for generating a prediction.

Additionally, hybrid classifiers were tested. Considering the large number of variables related to wavenumbers obtained by Raman spectroscopy, feature extraction and dimensionality reduction prior to feeding machine learning algorithms substantially improved the performance. First, Raman data were projected into an optimum number of latent vectors; redundant or irrelevant features were removed by projecting the data in a new reduced subset of latent vectors sorted by the amount of explained variance and extracted from combinations of the original features; then a second time PLS was applied to this new projected data such that most of the variance was captured and illustrated by the first two components; the classifier is then constructed in this new latent space;^[^ ^39^, ^40^ ^]^ this type of hybrid classifiers allows for meaningful graphical representations with no loss of any relevant information.

Due to the limited number of animals (*n* = 9), data splitting into calibration and validation datasets for model optimization was not useful. Instead, a leave‐one‐subject‐out cross‐validation (LOSOCV) method was implemented for predictive model construction and optimization. Similar to k‐fold cross‐validation, data was divided in groups. The characteristic of LOSOCV was that groups were formed with data from a single subject. Therefore, each subject was tested only once and one at a time, while the remaining subjects were used for training. Avoiding that data from the same pig were in different groups, prevents the model from overfitting and better simulates a real predictive situation of unknown data. Parameters were tuned according to the best averaged cross‐validated performance by selecting balanced accuracy as scoring metrics for evaluation.

The following three classification problems have been considered:

1. BC versus HI
2. BC versus HI_p_
3. BC versus HI versus HI_p_

All data analysis was performed in Spyder, a Python open source integrated development environment. For the analysis and development of predictive models, Sklearn, Scipy, Pandas, and Numpy libraries were mainly used, whereas Seaboorn and Matplotlib were applied to display plots and figures.

## Conflict of Interest

The authors declare no conflict of interest.

## Supporting information

Supporting Information

## Data Availability

The data that support the findings of this study are available on request from the corresponding author. The data are not publicly available due to privacy or ethical restrictions.
