# Supplementary material for: Photonic Technology for In Vivo Monitoring of Hypoxia–Ischemia
Source: Adv Sci (Weinh). 2022 Nov 15;10(1):2204834. doi: 10.1002/advs.202204834 (PMC9811478; doi:10.1002/advs.202204834)
Supplement: Supplementary file 1 — Supporting Information [file ADVS-10-2204834-s001.pdf]

Photonic Technology for *In Vivo* Monitoring of Hypoxia-Ischemia

Ion Olaetxea Hector Lafuente Eneko Lopez Ander Izeta Ibon Jaunarena Andreas Seifert**

Supporting Information

Supplementary tables

Table S1: Statistical significance of physiological parameters determined by Mann-Whitney U test between basal condition (BC) and hypoxia-ischemia (HI) condition and corresponding statistical significance given by the p-value and the effect size.

**Parameter**	**|U-statistic|**	**p-value**	**Effect size**
SO_2	0.0	<0.001*	1.0
pO_2	0.0	<0.001*	1.0
Lactate	1862.0	<0.001*	0.96
Base excess	6118.0	<0.001*	0.89
pH	6171.5	<0.001*	0.89
HCO_3^-	8218.5	<0.001*	0.85
TCO_2	9317.5	<0.001*	0.83
pCO_2	25688.0	<0.001*	0.54

Table S2: Statistical significance of physiological parameters determined by Mann-Whitney U test between basal condition (BC) and post-hypoxia-ischemia (HI_p) condition and corresponding statistical significance given by the p-value and the effect size.

**Parameter**	**|U-statistic|**	**p-value**	**Effect size**
Lactate	0.0	<0.001*	1.0
Base excess	0.0	<0.001*	1.0
pH	0.0	<0.001*	1.0
HCO_3^-	2.5	<0.001*	1.0
TCO_2	38.0	<0.001*	1.0
pCO_2	33258.0	<0.001*	0.47
pO_2	47549.5	<0.001*	0.24
SO_2	61415.0	<0.01*	0.02

Supplementary figures

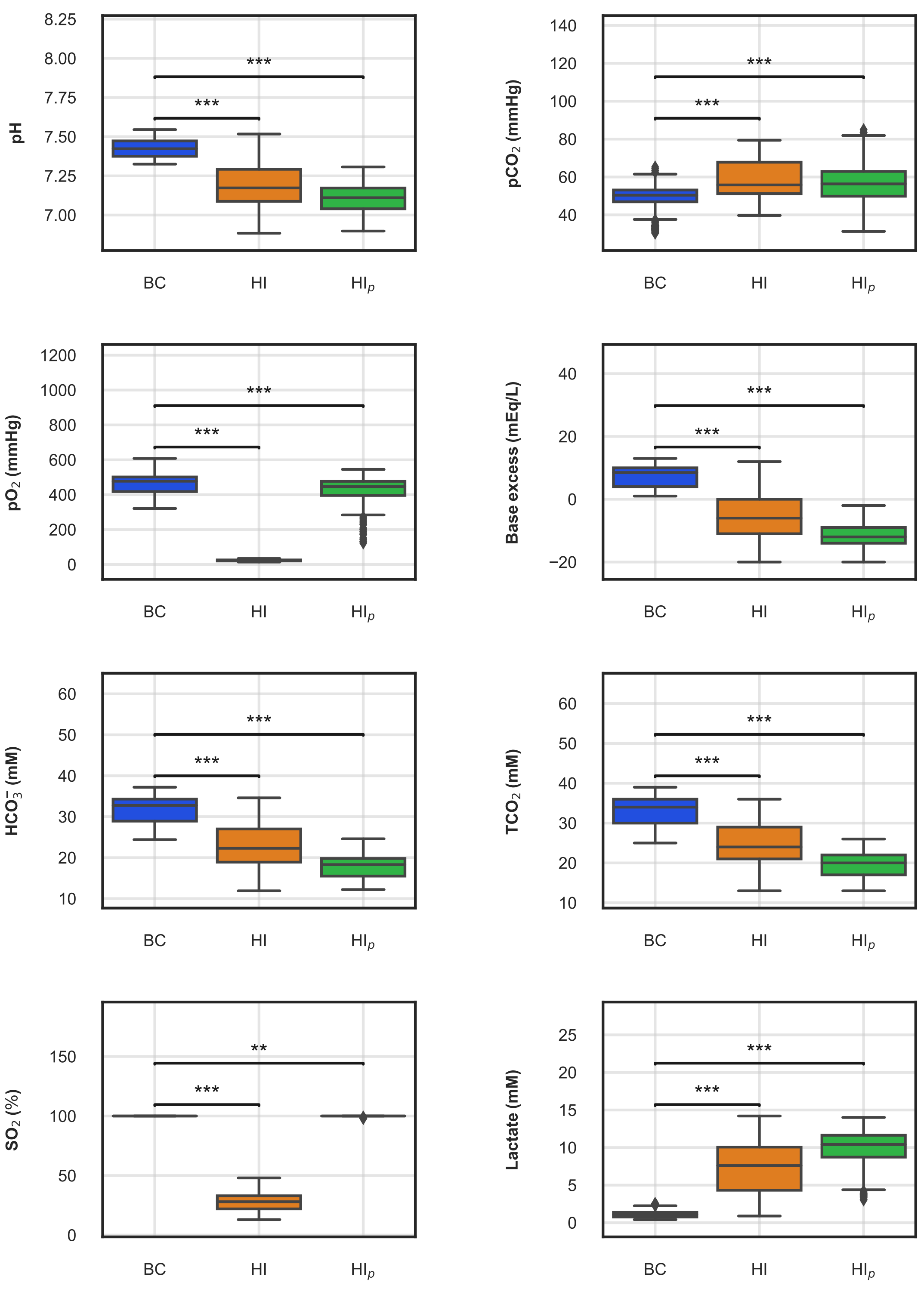

Figure S1: Exploratory analysis of basal condition (BC), hypoxia-ischemia event (HI) and post-hypoxia-ischemia (HI_p) groups for different physiological parameters. Box-and-whisker plot facilitates the comparison between the groups by displaying the distribution and skewness of the data.
Mann-Whitney U test
* $0.01 < \text{p-value} \leq 0.05$
** $0.001 < \text{p-value} \leq 0.01$
*** $\text{p-value} \leq 0.001$

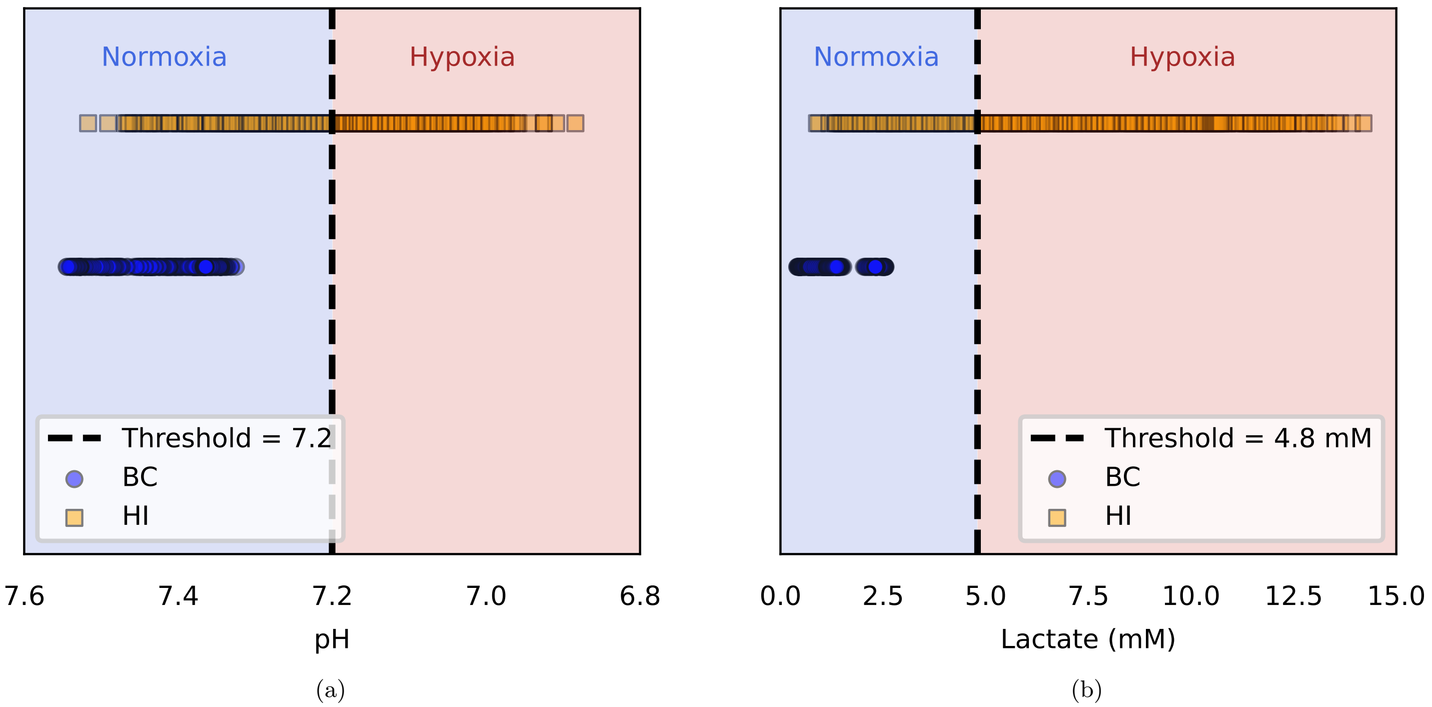

Figure S2: 1-D visual class separability between basal condition (BC) and hypoxia-ischemia (HI) based on standard clinical cut-off limits. **(a)** Decision region of binary classification (BC vs. HI) based on pH threshold of 7.2. **(b)** Decision region of binary classification (BC vs. HI) based on lactate concentration threshold of 4.8 mM.

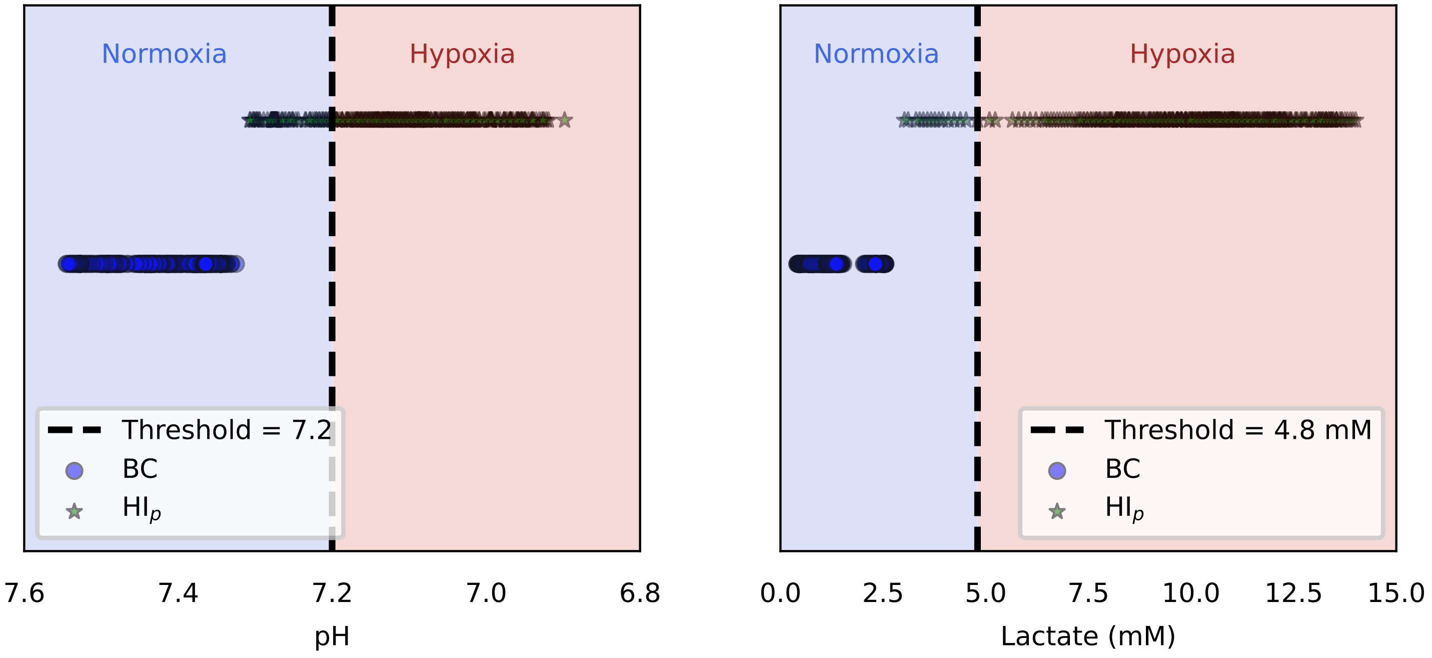

(a) (b)

Figure S3: 1-D visual class separability between basal condition (BC) and post-hypoxia-ischemia (HI_p) based on standard clinical cut-off limits. **(a)** Decision region of binary classification (BC vs. HI_p) based on pH threshold of 7.2. **(b)** Decision region of binary classification (BC vs. HI_p) based on lactate concentration threshold of 4.8 mM.

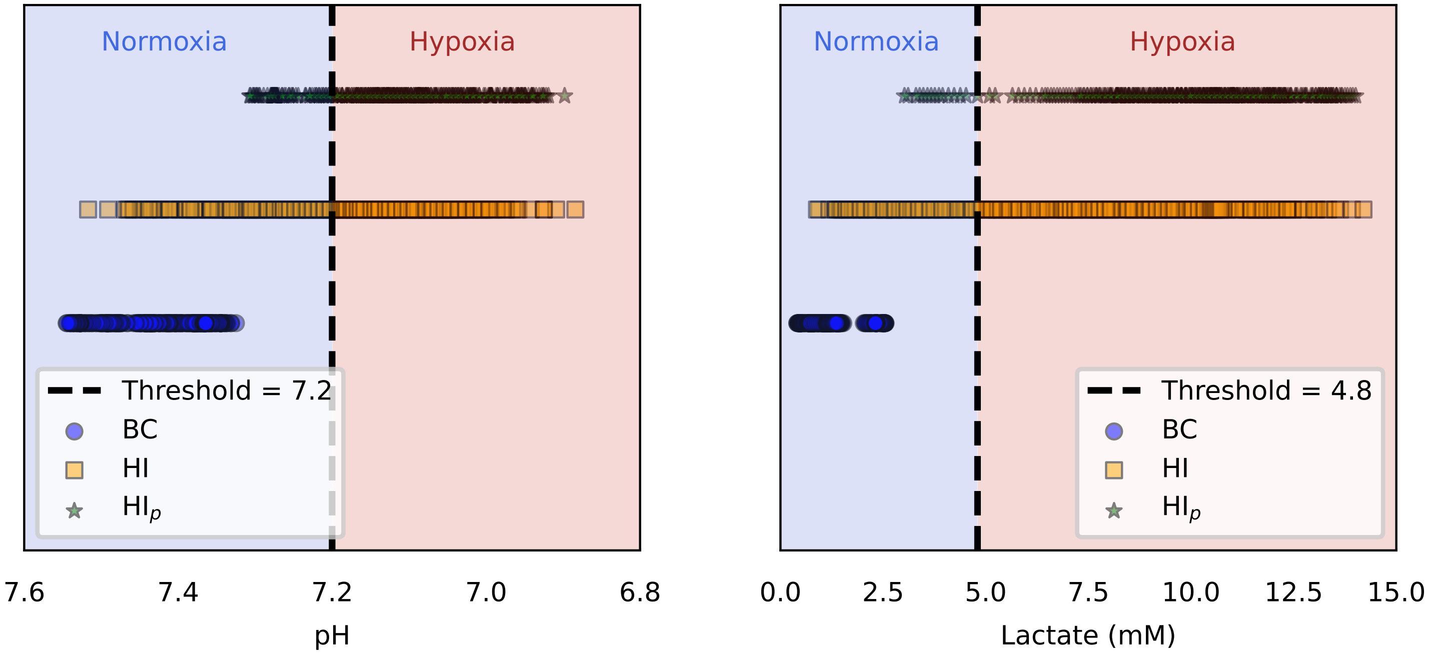

(a) (b)

Figure S4: 1-D visual multiclass separability between basal condition (BC), hypoxia-ischemia (HI) and post-hypoxia-ischemia (HI_p) based on standard clinical cut-off limits. **(a)** Decision region of binary classification (BC vs. HI vs. HI_p) based on pH threshold of 7.2. **(b)** Decision region of binary classification (BC vs. HI vs. HI_p) based on lactate concentration threshold of 4.8 mM.

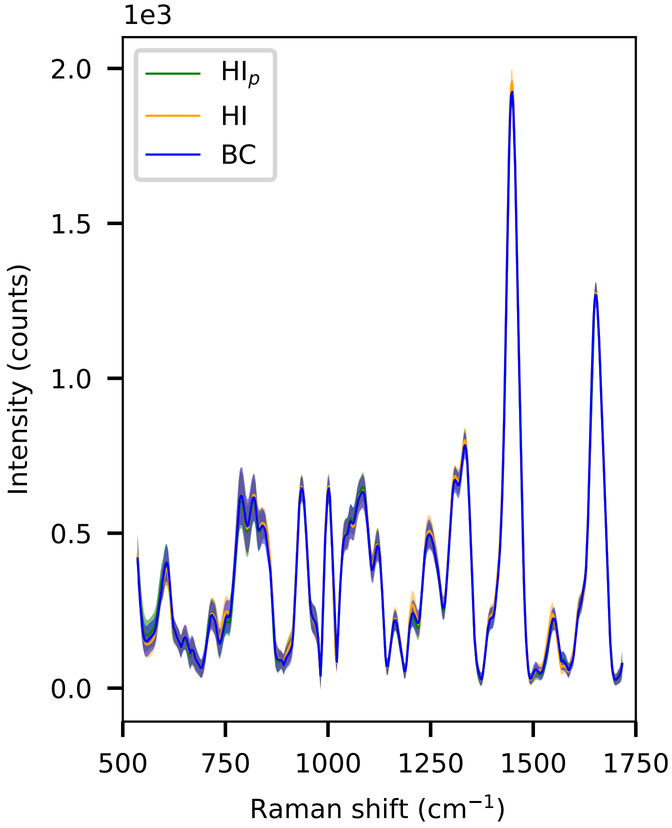

Figure S5: Averaged preprocessed Raman spectra from different clinical phases ± their standard deviation represented by the shaded area.

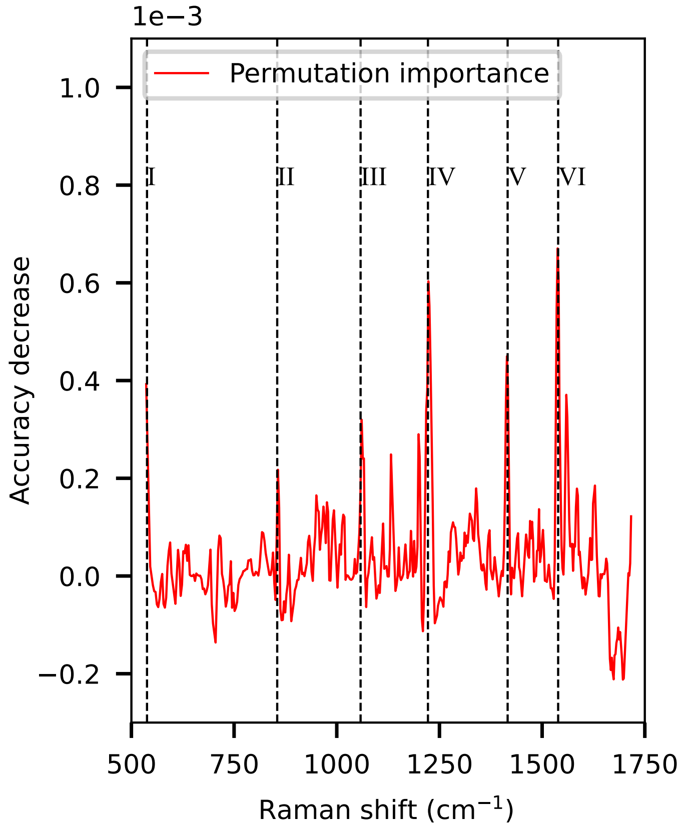

(a)

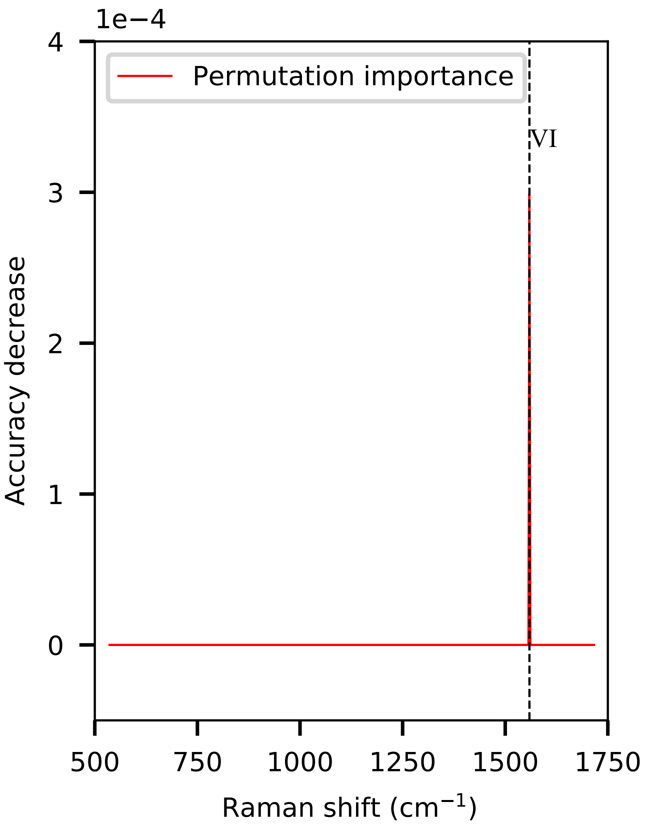

(b)

Figure S6: Permutation feature importance defined as accuracy decrease of fitted predictive models for BC vs. HI classification. **(a)** Averaged decrease of accuracy and standard deviation of fitted Partial Least Squares-Discriminant Analysis (PLS − DA) predictive models due to single parameter permutation. **(b)** Averaged decrease of accuracy and standard deviation of fitted Extreme Gradient Boosting (XGB) predictive models due to single parameter permutation. (Peaks: I. 543 cm^{-1}, II. 853 cm^{-1}, III. 1053 cm^{-1}, IV. 1222 cm^{-1}, V. 1420 cm^{-1}, VI. 1547 cm^{-1})

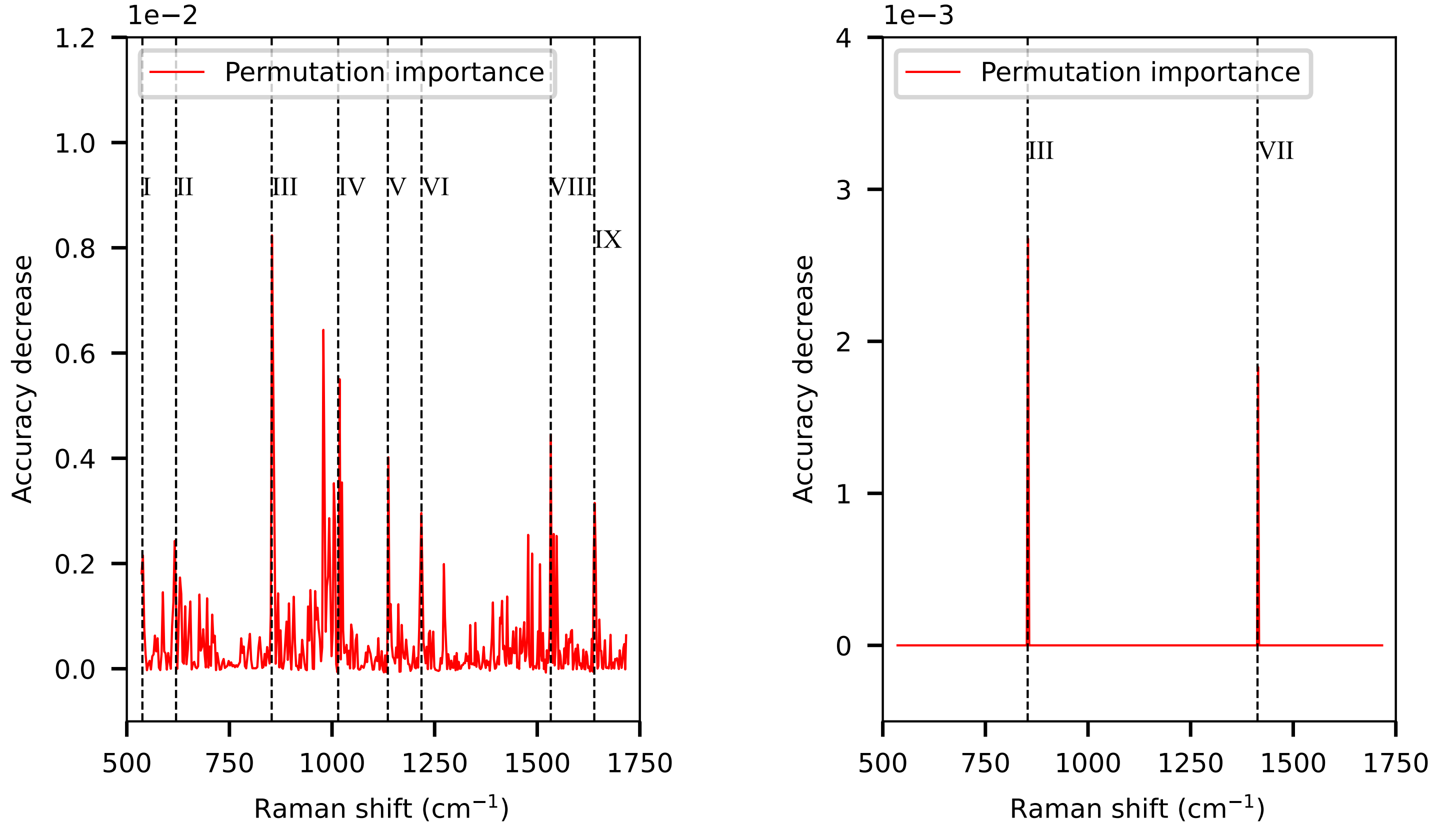

(a) (b)

Figure S7: Permutation feature importance defined as accuracy decrease of fitted predictive models for BC vs. HI_p classification. **(a)** Averaged decrease of accuracy of fitted Partial Least Squares-Discriminant Analysis (PLS − DA) predictive models due to single parameter permutation. **(b)** Averaged decrease of accuracy of fitted Extreme Gradient Boosting (XGB) predictive models due to single parameter permutation. (Peaks: I. 543 cm^{-1}, II. 620 cm^{-1}, III. 853 cm^{-1}, IV. 1005 cm^{-1}, V. 1125 cm^{-1}, VI. 1222 cm^{-1}, VII. 1420 cm^{-1}, VIII. 1547 cm^{-1}, IX. 1639 cm^{-1})

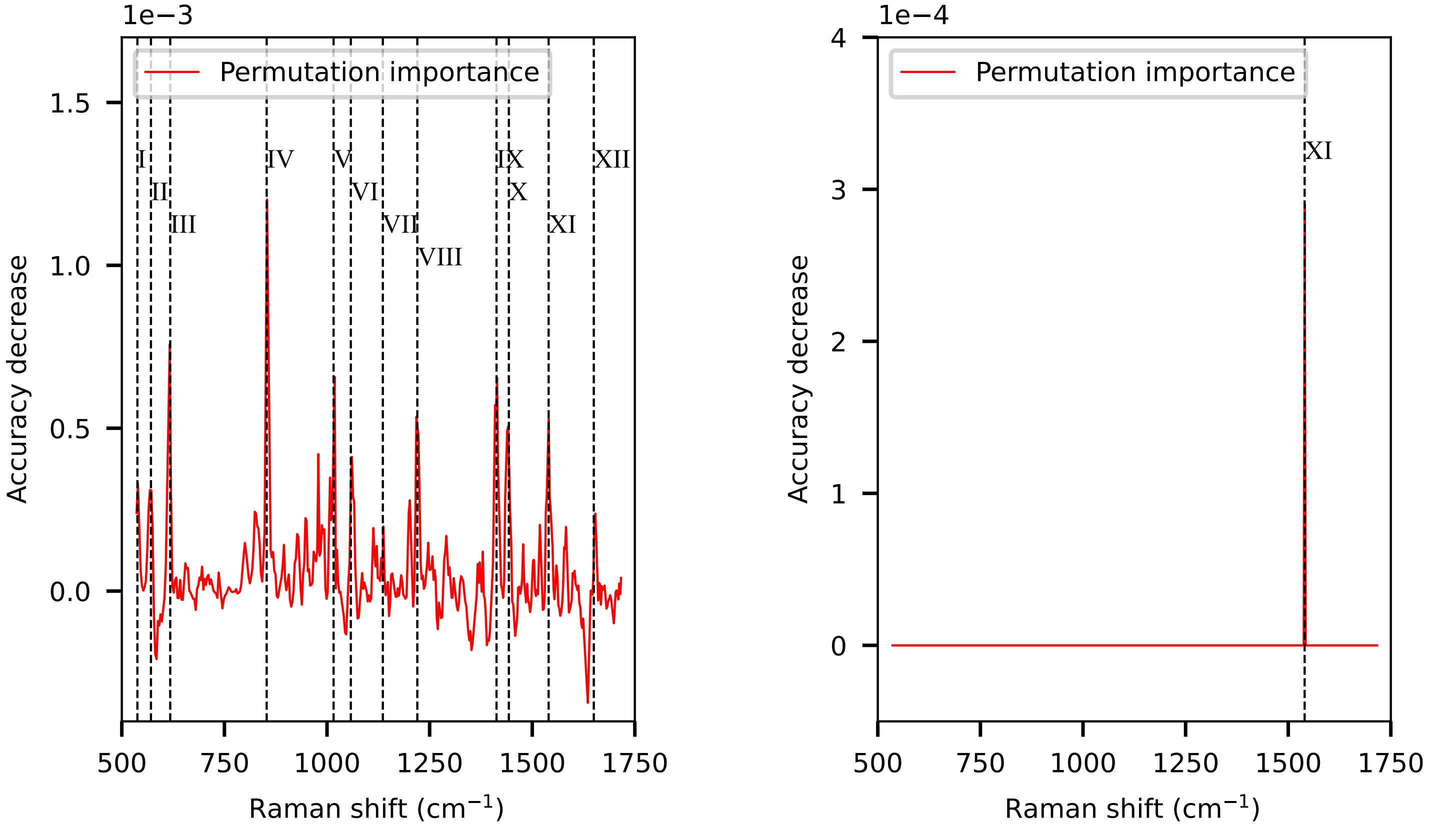

(a)

(b)

Figure S8: Permutation feature importance defined as accuracy decrease of fitted predictive models for BC vs. HI vs. HI_p classification. **(a)** Averaged decrease of accuracy of fitted Partial Least Squares-Discriminant Analysis (PLS − DA) predictive models due to single parameter permutation. **(b)** Averaged decrease of accuracy of fitted Extreme Gradient Boosting (XGB) predictive models due to single parameter permutation. (Peaks: I. 543 cm^{-1}, II. 571 cm^{-1}, III. 620 cm^{-1}, IV. 853 cm^{-1}, V. 1005 cm^{-1}, VI. 1053 cm^{-1}, VII. 1125 cm^{-1}, VIII. 1222 cm^{-1}, IX. 1420 cm^{-1}, X. 1459 cm^{-1}, XI. 1547 cm^{-1}, XII. 1639 cm^{-1})
